# Giant fecaloma in a 12-year-old-boy: a case report

**DOI:** 10.1186/1757-1626-2-127

**Published:** 2009-02-05

**Authors:** Juan D Garisto, Luis Campillo, Errol Edwards, Mireya Harbour, Rufino Ermocilla

**Affiliations:** 1 Department of Surgery, Regional Hospital of Changuinola, Bocas Del Toro, Apartado 801912090, El Dorado, Republic of Panama; 2 Pediatrics, Regional Hospital of Changuinola, Bocas Del Toro, Apartado 801912090, El Dorado, Republic of Panama; 3 Pathology, Regional Hospital of Changuinola, Bocas Del Toro, Apartado 801912090, El Dorado, Republic of Panama

## Abstract

**Background:**

Fecaloma is a mass of feces accumulated that is much harder in consistency than a fecal impactation. The aim of this report is to give a brief review of this entity and discuss the treatment options for these cases.

**Case presentation:**

We present the case of a 12-year-old boy who developed a fecaloma associated with chronic constipation. This is a rare case on a child which was treated by a sigmoid colectomy after failure of conservative measures of evacuation.

**Conclusion:**

Fecaloma should be considered in the differential diagnosis of patients with history of chronic constipation and abdominal mass.

## Background

Fecaloma is a mass of feces most frequently noted in the rectum and sigmoid, that is much harder than a fecal impactation due to coprostasis. Usually, the fecal matter accumulates in the intestine, then stagnates and increases in volume until the intestine becomes deformed and acquires characteristics similar to those of a tumor [1]. There are several causes of fecaloma and have been described in association with Hirschsprung's disease [2], psychiatric patients, Chagas disease, both inflammatory and neoplastic, and in patients suffering with chronic constipation. We are reporting a case of fecaloma of the sigmoid colon which is unusual in a child.

## Case presentation

We report a case of 12 years old boy, black, student who was admitted to our department for a 1 year progressive slowly growing of not painful abdominal mass occupying the right flank and right-lower quadrant. The patient had history of chronic constipation of 2 years referring 1 bowel movements every third-fourth day with hard stools. He denies symptoms of vomiting, nausea, diarrhea, fever, hematocquezia, hematuria, anorexia, lost weight. Not learning or mental disabilities were reported from his parents.

The patient was hospitalized previously four times during his life by history of constipation treated with laxatives and enemas and discharged with food recommendations. Only family history of diabetes in his grandmother.

On initial evaluation, the patient had a temperature of 37°C, blood pressure of 100/70 mm Hg, a heart rate of 85 beats per minute, a respiratory rate of 18 breaths per minute, with weight 30 kg and height 133 cms (BMI = 16.5, 18th centile).

Physical examination revealed a good general condition, cardiac and lungs examination were unremarkable. Abdomen without scars, soft, not distended, tympanic in 4 quadrants, normoactive bowel sound, palpation revealing an abdominal mass smooth, mobile, nontender in the lower right flank and quadrant of ± 13 cm of diameter. Rectal examination revealed no palpable stool in the rectal vault and was hemoccult negative. No malformations were finding during the examination.

The laboratory test parameter during admission were: hemoglobin 13.2 g/dl, hematocrit of 39.5%, WBC 5.500, platelets 374.000 mm3; creatinine 0.8/BUN 70, Na:142, K:4.3, urinalysis and liver function tests were normal. Stool samples for parasites were negative.

**Figure 1:**
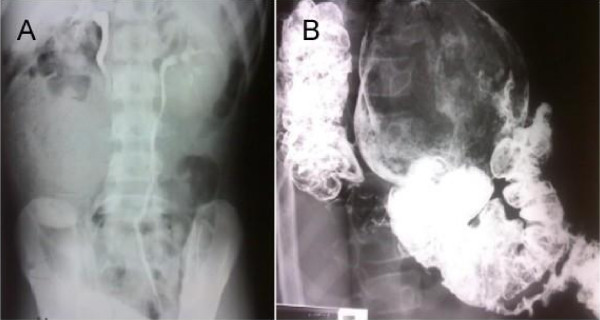
**A. Intravenous pyelogram shows large fecaloma compressing the right ureter**. B. Barium enema demonstrates the calcified fecaloma in the sigmoid.

Intravenous pyelogram revealed a large rounded mass of approximately 12 cm. in diameter at the colon which seemed to have an irregular mottled texture. The right ureter seemed to be somewhat displaced by the mass that produce a slightly enlargement of the renal pelvis (Fig. 1-A). Roentgenologic examination of the colon after barium enema showed an intraluminal mass having a smooth contour and without any mucosal attachment (Fig. 1-B).

Ultrasound of the abdomen showed a mass arising probably from the intestine with no evidence of hydronephrosis.

The flexible rectosigmoidoscopy was done and only passed through 25 cm of rectum which presented a normal rectum with a roomy sigmoid colon and a large, firm fecal ball. Sigmoidoscopic rectal biopsies were not performed and fragmentation was attempted but abandoned because of pain during the procedure. Repeated enemas and laxatives were unsuccessful in evacuating the fecal mass.

The patient underwent elective laparotomy. Preoperative bowel cleaning was performed with phosphosoda solution and lavage with warm saline until evacuations were liquid without fecal residue. A 20-cm midline incision (infraumbilical with supraumbilical extension) was made. During the procedure we founded an enlarged sigmoid colon lateralized to the right showing a large, rock-hard mass, smooth and ovoid in shape, measuring about 14 cm, which appeared to be a hardened mass of feces. The rest of the colon appeared normal and empty.

**Figure 2:**
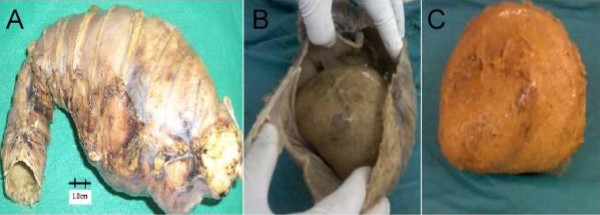
**A. Sigmoid colon resected**. B. Macroscopic specimen after incision of the intestinal wall showing the fecaloma. C. Fecaloma measuring 12 cm of length.

The sigmoid colon with the mass was resected (Fig. 2-A) and an end-to-end anastomosis was done. The patient recovered for the surgical procedure and had an uneventful convalescence. After six months our patient refers 1 bowel movement per day performing soft stools with no recurrence of his symptoms leading a completely normal healthy life.

**Figure 3:**
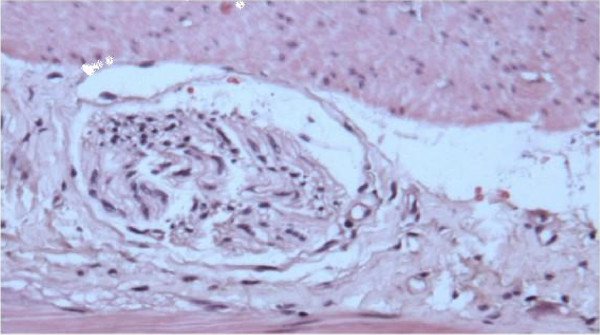
**The microscopic view of the myenteric (Auerbach) plexus shows extensively the presence of hyperrophied, disorganized and abundant nerve fibers with the absence of ganglion cells**.

Examination of the gross specimen after incision of the intestinal wall showed a firm fecal mass covered with a white material (barium) which filled the lumen but was unattached to the wall of the colon (Fig. 2-B–C). No lesions were observed at the sigmoid wall. Microscopic examination of the colon demonstrates normal intestinal mucosa without ulcers. Myenteric plexus shows nerve fibers with lack of ganglion cells (Fig. 3).

## Discussion

Fecal impactation is a common condition and fecaloma is an extreme variety of impactation that refers to an accumulation of fecal material which forms a mass separable from the rest of the bowel contents. This condition is uncommon and the majority of reported cases have been in adults. Symptoms of fecaloma are usually nonspecific with "overflow "type of diarrhea, constipation, weight loss and vague abdominal discomfort after meals. Constipation was the main symptom referred by our patient. In fact, constipation is one of the most frequently experienced gastrointestinal complaints and one of the most frequent indications for medical consultation [3].

The composition of the mass is quite inconstant, but usually consists of fecal matter and intestinal debris [4]. Often is formed in a laminated fashion due to deposits of calcium soaps in layers. Distal colon and rectum are the most common sites for fecalomas [5]. Common complications of fecalomas and fecal impaction include obstruction [6], perforation [7,8], ulceration [9] and hydronephrosis [10,11]. Most of the fecal impactations are successfully treated by conservative methods such as laxatives, enemas and digital evacuation [12]. Another approach such as endoscopic removal had also been described [13]. When conservative measures have failed, as in this case, a surgical intervention may be needed for removal of a fecaloma and prevent complications.

In our case, ganglion cells were not found in the myenteric plexus in the affected part of the colon histopathologically, and nerve trunks in this part were both hyperthrophied and increased in number. However, these primary abnormalities were probably due to a lack of migration of the neuroblast into the alimentary tract during the embryologic development. This alteration can be attributed as the main factor to develop a fecaloma in our patient.

## Conclusion

Fecaloma should be considered in the differential diagnosis of any patient with history of chronic constipation and abdominal mass. Often the diagnosis can be made form the clinical and radiologic features. In the beginning, therapy should be conservative. Rarely laparotomy is required to remove the mass.

## Competing interests

The authors declare that they have no competing interests.

## Authors' contributions

JGR was a major contributor in writing the manuscript. EE and LC were the surgeons that perform the laparotomy with JGR. TH with LC were the attendings for the department of pediatrics and surgery that follows the patient after discharge. RE performed the histological examination of the sigmoid colon. All authors read and approved the final manuscript.

## Consent

Written informed consent was obtained from the patient's mother for publication of this case report and accompanying images. A copy of the written consent is available for review by the Editor-in-Chief of this journal.
